# Supplementary material for: Muscle progenitor specification and myogenic differentiation are associated with changes in chromatin topology
Source: Nat Commun. 2020 Dec 4;11:6222. doi: 10.1038/s41467-020-19999-w (PMC7718254; doi:10.1038/s41467-020-19999-w)
Supplement: Supplementary file 1 — Supplementary Information [file 41467_2020_19999_MOESM1_ESM.pdf]

SupFig.1

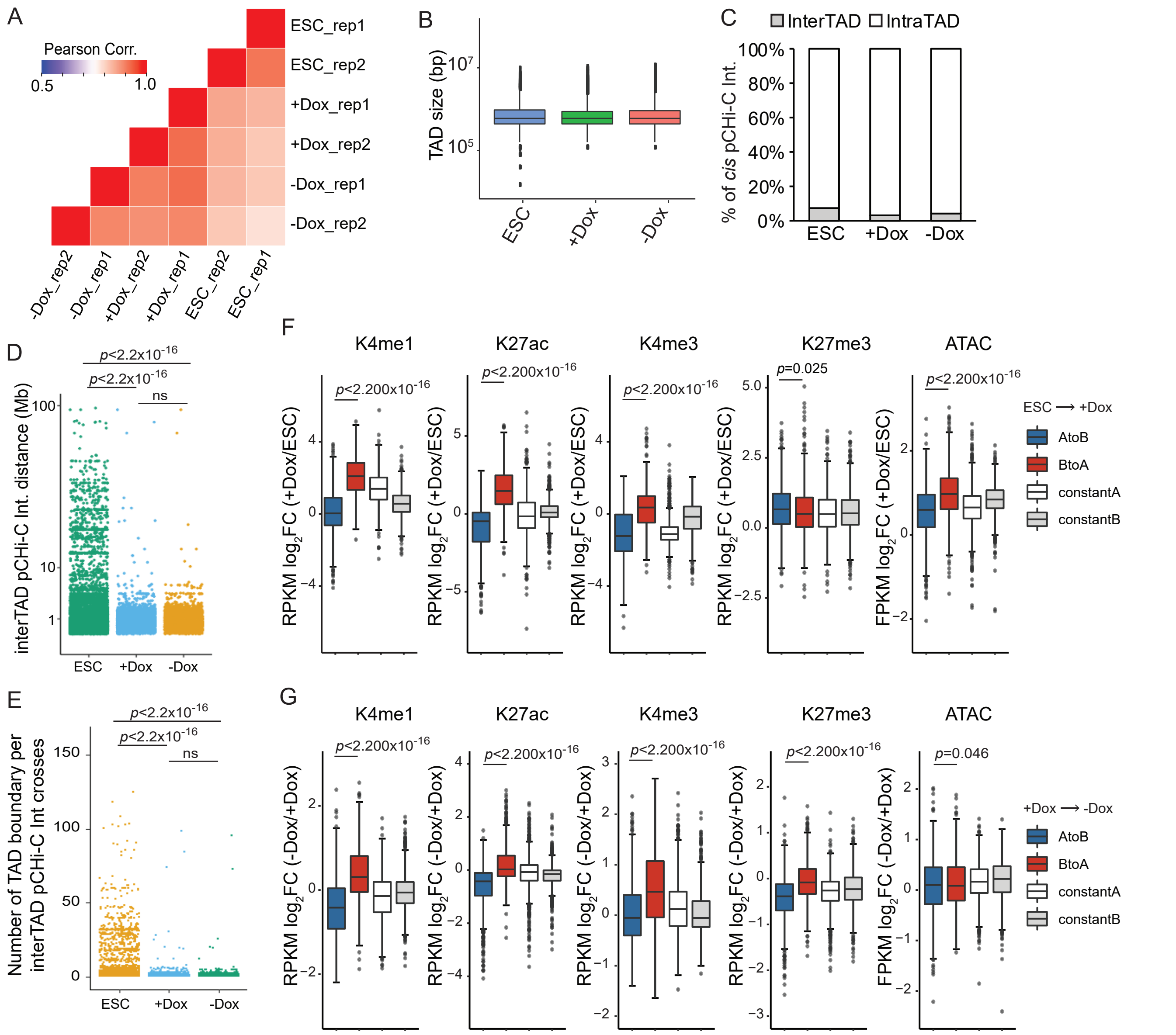

Supplementary Figure 1. Chromatin conformation is altered during muscle progenitor specification.

(A) Pairwise Pearson correlations of HiC matrices between replicates and across ESC, +Dox and –Dox iPax7 populations. (B) TAD size distribution in ESC (n=2,837) and iPax7 (+Dox (n=2,841) and –Dox (n=2755)) ESC and iPax7 cells. The boxes denote the 25th and 75th percentile (bottom and top of box), and median value (horizontal band inside box). The whiskers indicate the values observed within up to 1.5 times the interquartile range above and below the box. (C) Quantification of inter-TAD pCHi-C interactions among all high-confidence cis-pCHi-C interactions. n=106,742 for ESCs, n=121,266 for iPax7 cells that were Dox-treated (+Dox), and n=113,601 for cells after Dox withdrawal (-Dox). (D) Distance distribution of high-confidence inter-TAD pCHi-C interactions in ESC (n=7,467) and iPax7 (+Dox (n=3,911) and –Dox (n=3,883)) and iPax7 cells. Asterisks indicate statistical significance (p<0.0001, two-tailed Student's t-test). (E) Quantification of TAD boundaries crossed in ESC (n=2816) and iPax7 cells (+Dox, n=2,821; –Dox, n=2,734) and iPax7 cells (per inter-TAD pCHi-C interaction). Asterisks indicate sStatistical significance (p<0.0001, tested with two-tailed Student's t-test). (F, G) Log2 fold-change (FC) of signal enrichment of ChIP-seq (H3K27Ac, H3K4me1 H3K4me3 and H3K27me3) and ATAC-seq for compartments that switch (blue: A to B, red: B to A) or remain unchanged (white: constant A, grey: constant B) in +Dox iPax7 vs. ESC and -Dox vs. +Dox iPax7comparing ESC to iPax7 progenitors (+Dox) (A to B: n=811; B to A: n=740; constant A: n=878; constant B: n=933) (F) and iPax7 progenitors to differentiated iPax7 cells (-Dox) (A to B: n=755; B to A: n=504; constant A: n=1,005; constant B: n=1,044) (G) comparisons. The boxes denote the 25th and 75th percentile (bottom and top of box), and median value (horizontal band inside box). The whiskers indicate the values observed within up to 1.5 times the interquartile range above and below the box. Statistical significance tested with two-tailed Student's t-test. Asterisks indicate statistical significance (p<0.0001, two-tailed Student's t-test).

SupFig.2

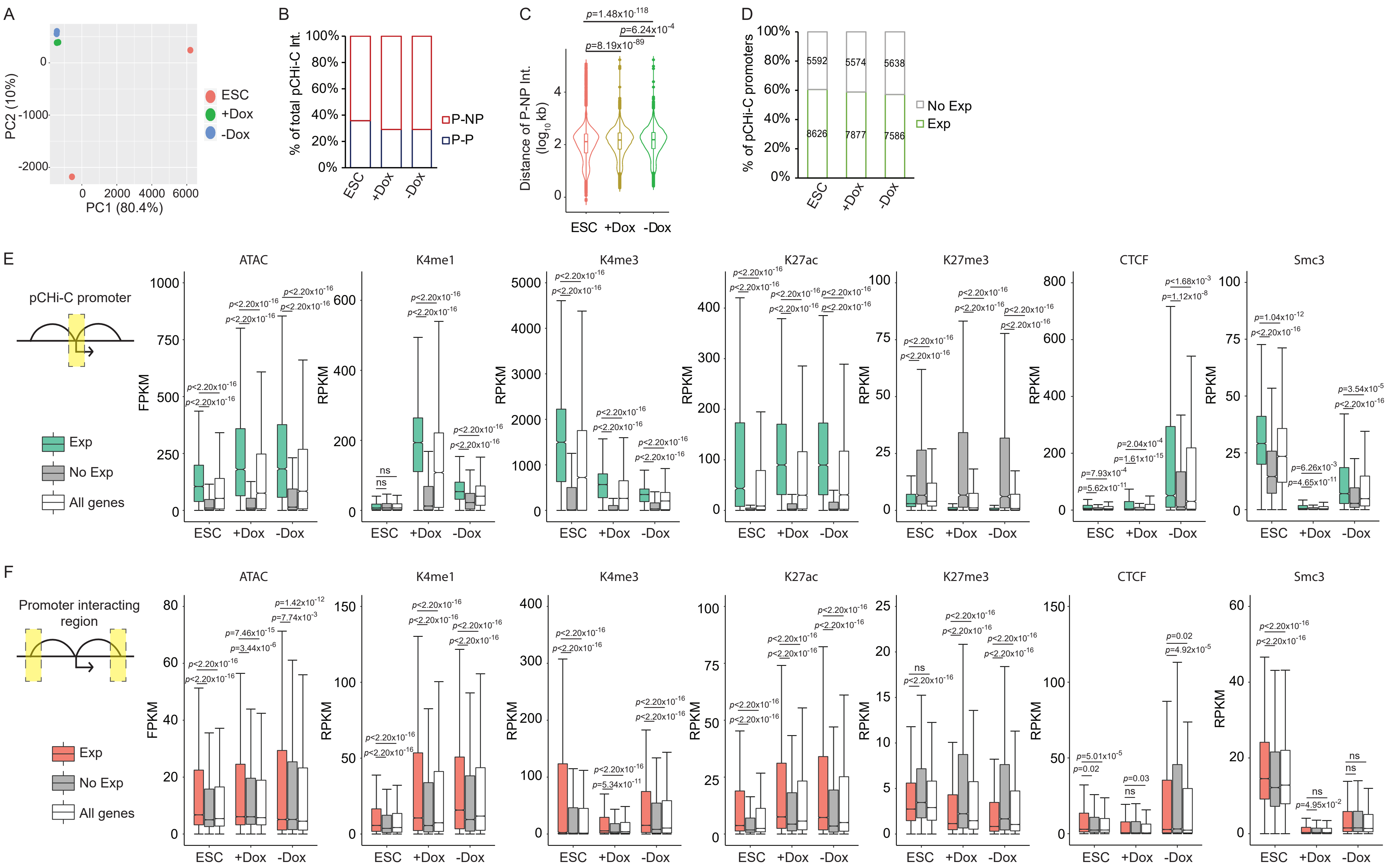
A
PC2 (10%)
PC1 (80.4%)
ESC
+Dox
-Dox
B
% of total pCHi-C Int.
P-NP
P-P
C
Distance of P-NP Int. (log10 kb)
p=1.48x10-118
p=6.24x10-4
p=8.19x10-89
D
% of pCHi-C promoters
No Exp
Exp
5592
5574
5638
8626
7877
7586
E
pCHi-C promoter
Exp
No Exp
All genes
ATAC
K4me1
K4me3
K27ac
K27me3
CTCF
Smc3
FPKM
RPKM
F
Promoter interacting region
Exp
No Exp
All genes
ATAC
K4me1
K4me3
K27ac
K27me3
CTCF
Smc3
FPKM
RPKM

Supplementary Figure 2. Comparing pCHi-C results in mouse ESC and iPax7 muscle cells.
(A) CHiCAGO score-based PCA analysis of replicates of pCHi-C interactions detected in ESC, Dox-treated (+Dox) and untreated (-Dox) iPax7 cells. (B) Quantification of high-confidence promoter to promoter (P-P) and promoter to non-promoter (P-NP) pCHi-C interactions in ESC (n=99,191), +Dox (n=110,935) and -Dox (n=103,510) iPax7 populations. (C) Distance distribution for P-NP (ESC: n=68,279; +Dox: n= 85,550; -Dox: n= 79,993) interactions in each cell population. The boxplot within each violin plot shows the 25th and 75th percentile (bottom and top of box), and median value (horizontal band inside box). The whiskers indicate the values observed within up to 1.5 times the interquartile range above and below the box. Statistical significance tested with two-tailed Student's t-test. interquartile range, and whiskers show 5th and 95th percentiles. Statistical significance determined by Mood's median test. (D) Quantification of pCHi-C captured promoters based on gene expression in each cell population. Exp: expressed gene. No Exp: non-expressed gene. (E, F) Signal enrichment of ATAC-seq and ChIP-seq at pCHi-C promoter regions (baits) (E) and promoter-interacting regions (non-baits) (F) for expressed genes (Exp) and non-expressed genes (No Exp). Quantification for all genes with pCHiC interactions in each cell population are shown for comparison. For promoter regions of expressed genes in (E), n=8,626 for ESC, n=7,877 for +Dox and n=7,586 for –Dox. For promoter regions of non-expressed genes in (E), n=5,592 for ESC, n=5,574 for +Dox and n=5,638 for –Dox. For All genes in (E), n=14,218 for ESC, n=13,451 for +Dox and n=13,224 for –Dox. For promoter-interacting regions of expressed genes in (F), n=30,855 for ESC, n=45,687 for +Dox and n=44,018 for –Dox. For promoter-interacting regions of non-expressed genes in (F), n=21,947 for ESC, n=27,228 for +Dox and n=25,585 for –Dox. For All genes in (F), n=43,163 for ESC, n=61,314 for +Dox and n=58,758 for –Dox. The boxes denote the 25th and 75th percentile (bottom and top of box), and median value (horizontal band inside box). The whiskers indicate the values observed within up to 1.5 times the interquartile range above and below the box. Statistical significance tested with two-tailed Student's t-test. Asterisks indicate statistical significance (* $p<0.05$, ** $p<0.001$, *** $p<0.0001$, two-tailed Student's t-test).

SupFig.3

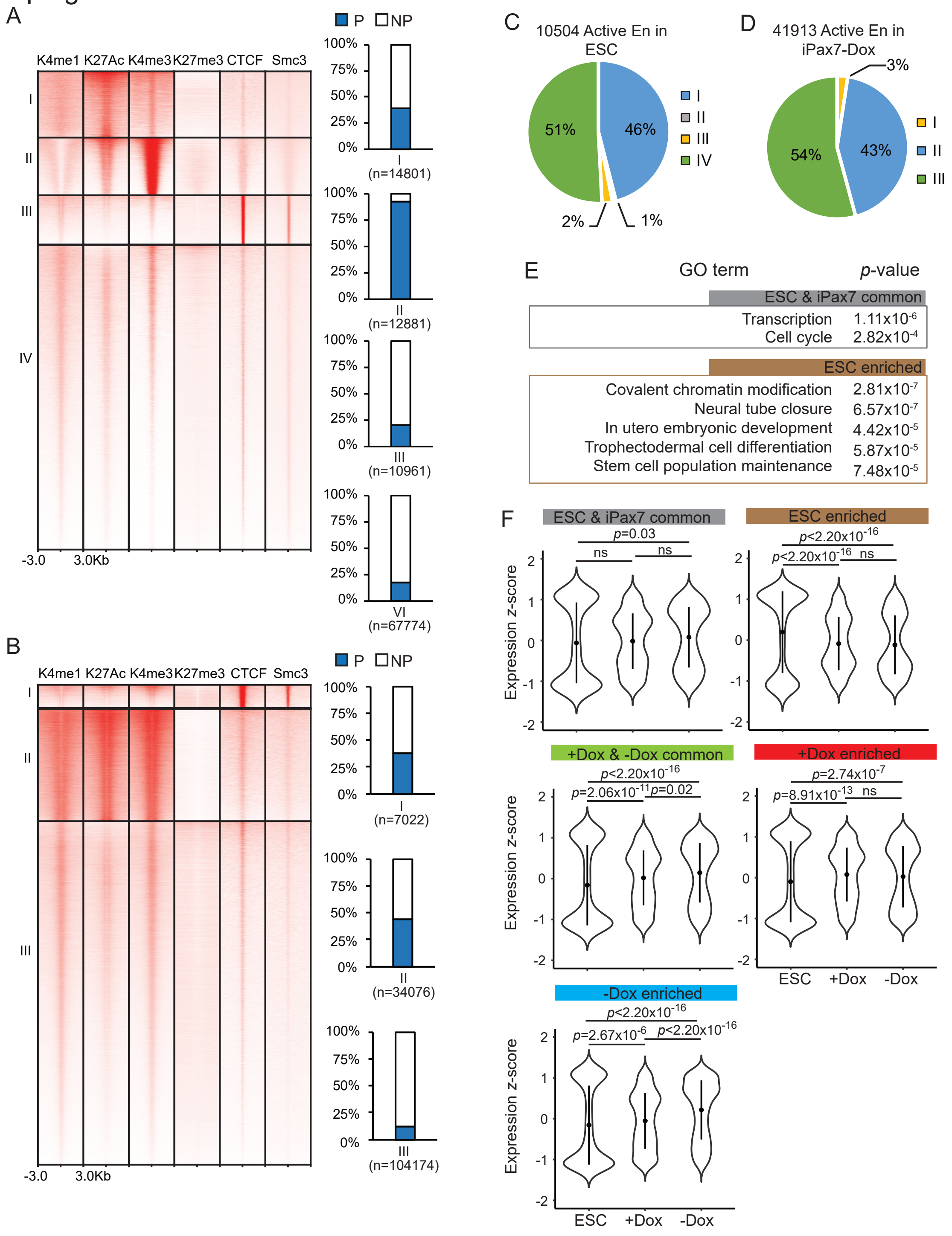

Supplementary Figure 3. Lineage specificity of pCHiC interactions.
(A, B) Annotations of open chromatin regions in ESC (A) and -Dox iPax7 cells (B). Open chromatin regions in the two cell populations were detected by ATAC-seq and then classified with k-means clustering on the basis of epigenomic features and co-localization with CTCF and Smc3 ChIP-seq enrichment. Each group (I-IV for ESC and I-III for –Dox iPax7 cells) of open chromatin regions was further annotated with genic features based on the location of peaks within the group, and the result was presented as a bar plot on the right. P, promoter; NP, non-promoter region. (C) Quantification of active enhancers detected in ESC from group I-IV in (A). (D) Quantification of active enhancers detected in –Dox iPax7 cells from group I-III in (B). (E) GO analysis for genes with high-confidence pCHi-C interactions from ESC and iPax7 common- and ESC-enriched clusters from Fig. 2D. (F) Expression z-scores for genes from the five P-En interaction clusters in the heat map of Fig. 2D. ESC & iPax7 common: n=1,313; ESC enriched: n=2,056; +Dox & -Dox common: n=1,785; +Dox enriched: n=3,019; -Dox enriched: n=3,030. Mean z-score and sd are represented with dot and error bar, respectively. Asterisks indicate statisticalStatistical significance (* p<0.05, ** p<0.001, *** p<0.0001,tested with two-tailed Student’s t-test).

SupFig.4

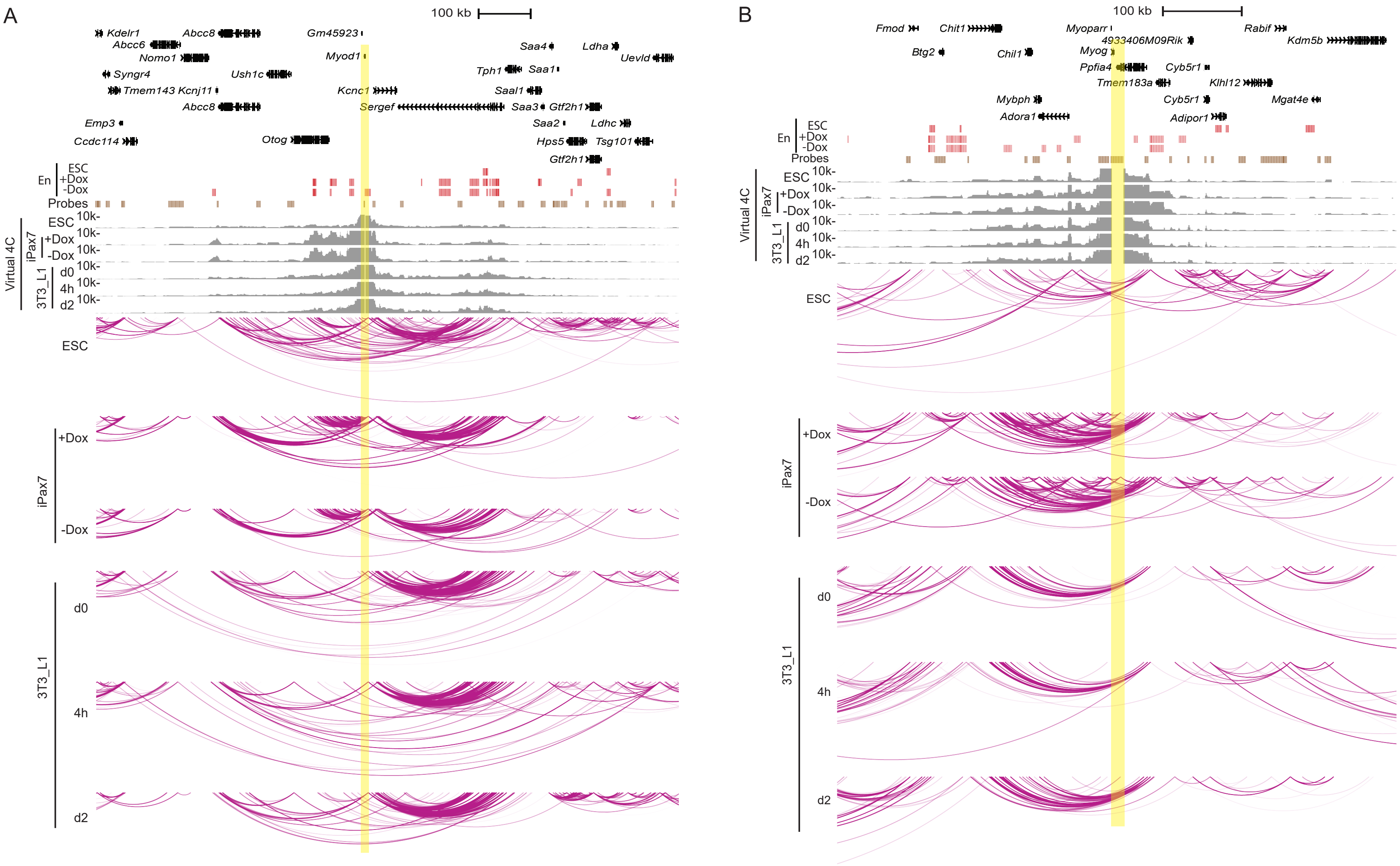

Supplementary Figure 4. pCHiC interactions at Myod1 and Myog loci.
(A, B) Examples of high-confidence pCHi-C interactions around Myod1 (A) and Myog gene clusters (B) in ESC, iPax7, and 3T3-L1 cells. For 3T3-L1 cells, data for day 0 (d0), 4 hours (4h) and day 2 (d2) of differentiation are shown. Panel A shows an expanded view of data also shown in Fig. 2E. Myod1 and Myog genes are highlighted in yellow.

SupFig.5

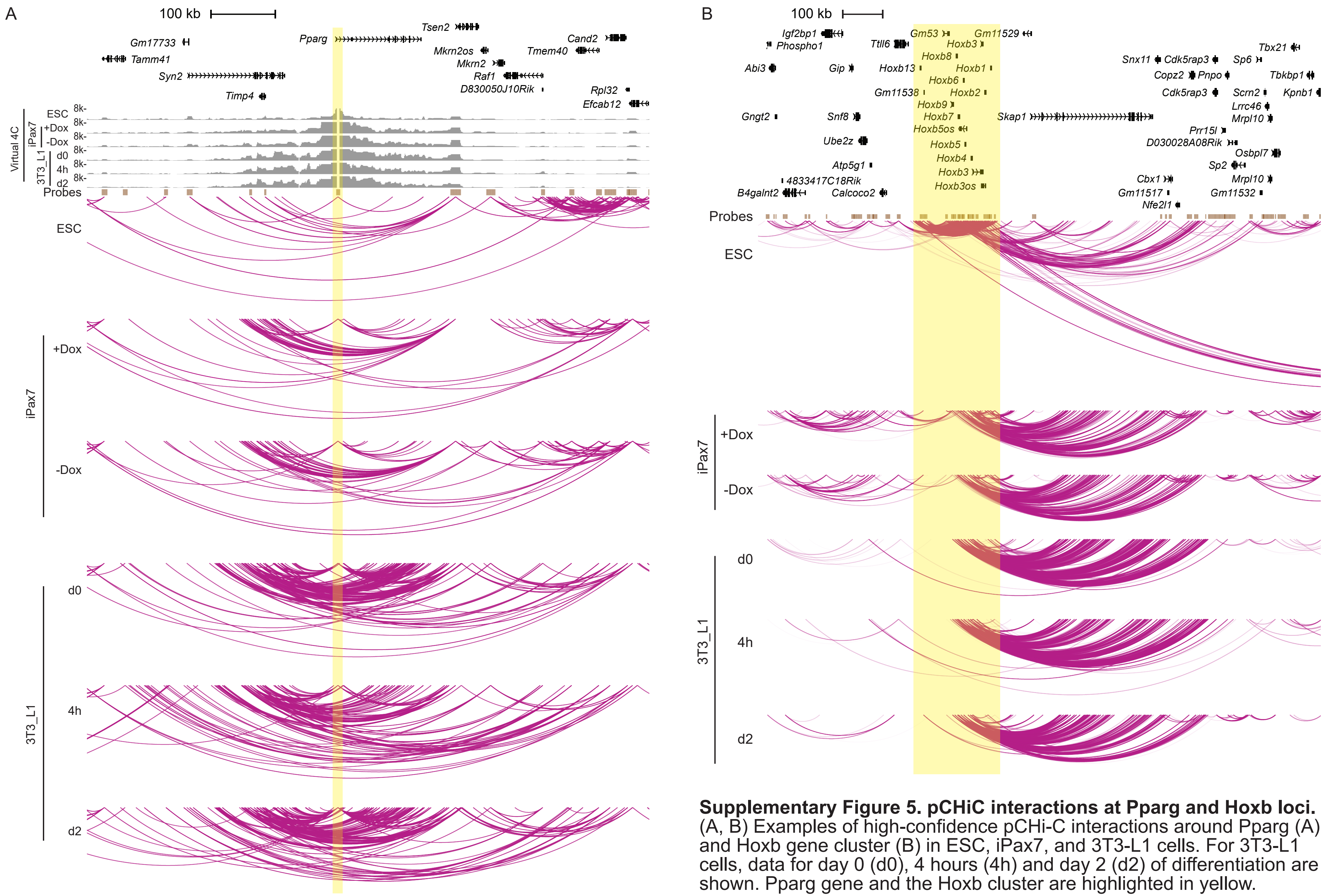

Supplementary Figure 5. pCHiC interactions at Pparg and Hoxb loci. (A, B) Examples of high-confidence pCHi-C interactions around Pparg (A) and Hoxb gene cluster (B) in ESC, iPax7, and 3T3-L1 cells. For 3T3-L1 cells, data for day 0 (d0), 4 hours (4h) and day 2 (d2) of differentiation are shown. Pparg gene and the Hoxb cluster are highlighted in yellow.

SupFig.6

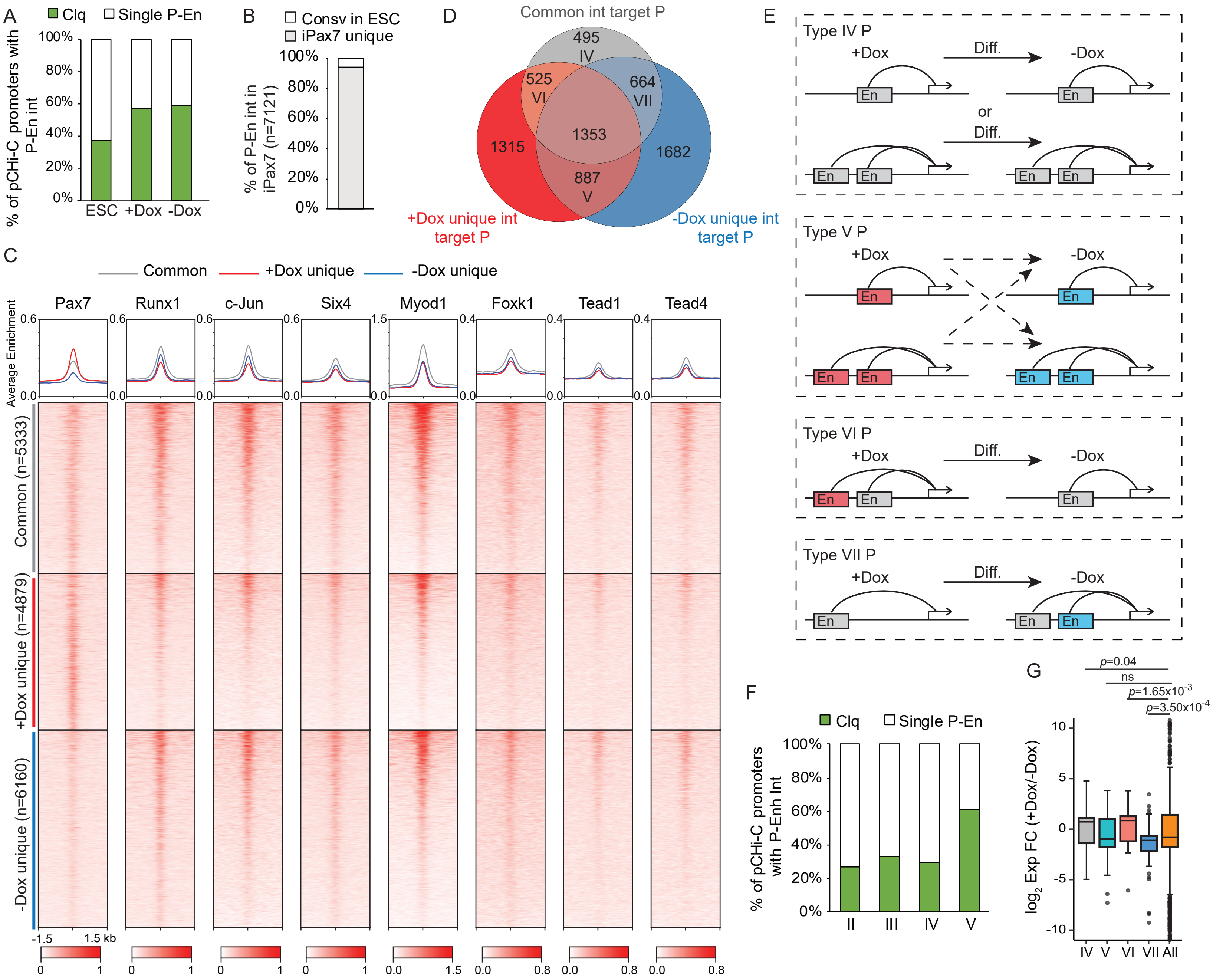
A
Clq
Single P-En
% of pCHi-C promoters with P-En int
100%
80%
60%
40%
20%
0%
ESC +Dox -Dox
B
Consv in ESC
iPax7 unique
% of P-En int in iPax7 (n=7121)
D
Common int target P
495 IV
525 VI
664 VII
1353
1315
1682
887 V
+Dox unique int target P
-Dox unique int target P
E
Type IV P
+Dox
Diff.
-Dox
En
or
Type V P
Type VI P
Type VII P
C
Common
+Dox unique
-Dox unique
Pax7 Runx1 c-Jun Six4 Myod1 Foxk1 Tead1 Tead4
Average Enrichment
Common (n=5333)
+Dox unique (n=4879)
-Dox unique (n=6160)
-1.5
1.5 kb
F
Clq
Single P-En
% of pCHi-C promoters with P-Enh Int
II III IV V
G
p=0.04
ns
p=1.65x10-3
p=3.50x10-4
log2 Exp FC (+Dox/-Dox)
IV V VI VII All

Supplementary Figure 6. Characterizing differentiation-dependent P-En interactions during iPax7 muscle progenitor differentiation.

(A) Quantification of pCHi-C promoters in P-En cliques (Clq). P: promoter. En: enhancer. ESC: n=2,574; +Dox: n=5,239; -Dox: n=5,606. (B) Quantification showing that a small percentage of pCHi-C captured high-confidence P-En interactions in iPax7 cells (either with or without Dox treatment, n=7,121) are conserved from ESC. (C) Enrichment profiles (top) and heat maps (bottom) of TF ChIP-seq at active enhancers detected in iPax7 cells from common, +Dox unique and -Dox unique P-En interactions. (D, E) Classification of enhancer contacted promoters (P) according to the three rewiring modes of interactions in Fig. 3 (B) during iPax7 cell differentiation. Promoters were grouped into seven types (labeled I-VII), and the number of promoters found in each type is denoted in the Venn diagram (D). Schematics of rewiring of P-En interactions during differentiation for type IV-VII promoters are shown in (E) with common enhancers in grey, +Dox specific enhancers in red, and –Dox specific enhancers in blue. (F) Quantification of type II-V pCHi-C promoters (Fig. 3C and Supplementary Fig. 6D) that interact with single or >2 enhancers (Clq). II: n=1,315; III: n=1,682; IV: n=495; V: n=887. (G) Transcriptional changes for genes with type IV-VII pCHi-C promoters grouped in (D) during iPax7 progenitor differentiation. Only differentially expressed genes (padj<0.05 from DESeq2) are included (IV: n=58; V: 133; VI: 75; VII: n=106). The complete set of differentially expressed genes with enhancer interactions during iPax7 cell differentiation was used as a control (All, n=2,831). The boxes denote the 25th and 75th percentile (bottom and top of box), and median value (horizontal band inside box). The whiskers indicate the values observed within up to 1.5 times the interquartile range above and below the box. Statistical significance tested with two-tailed Student's t-test. Statistical significance determined with two-tailed Student's t-test. * p<0.05, ** p<0.001.

SupFig.7

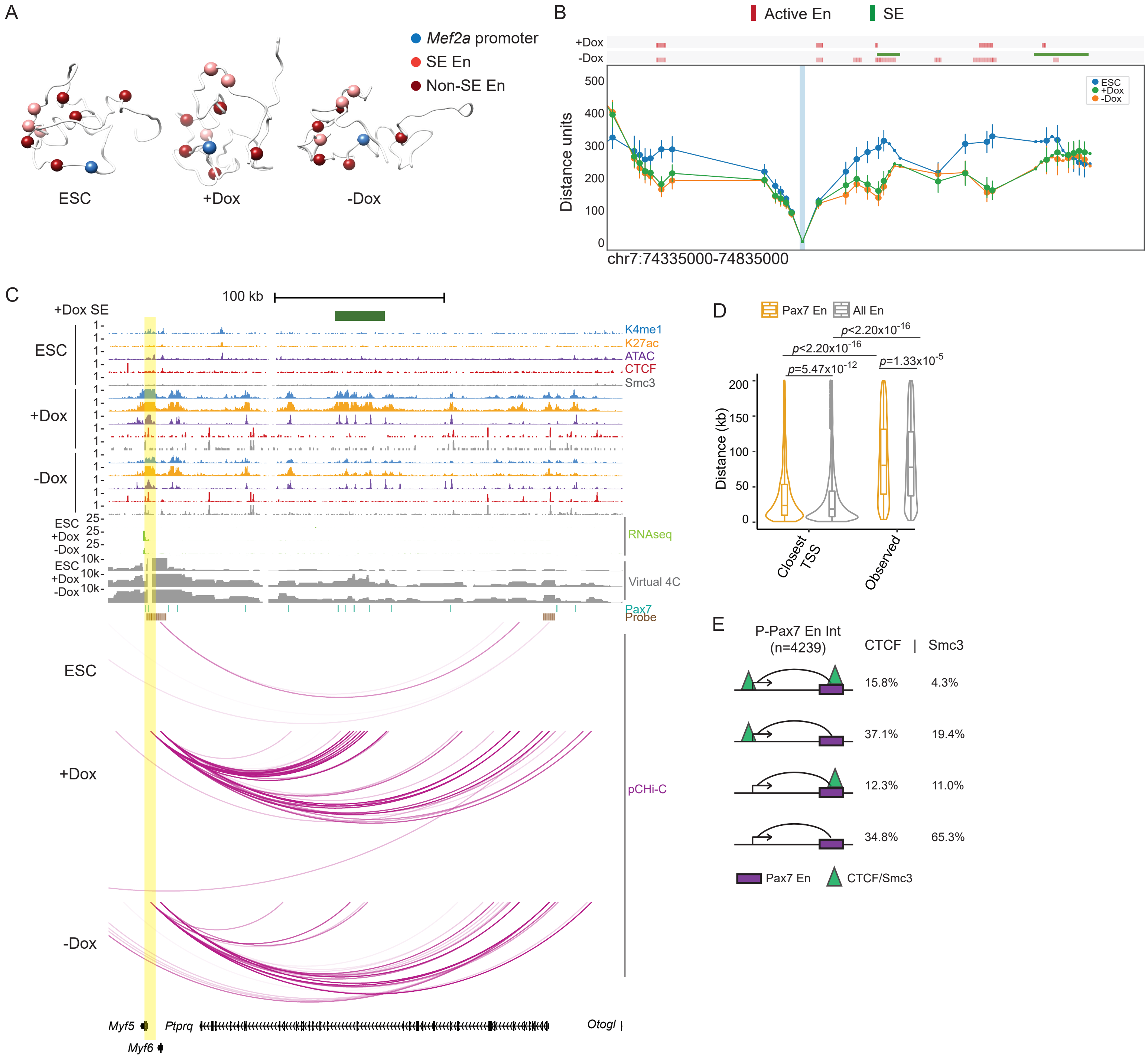

Supplementary Figure 7. Muscle cell-specific topology of Mef2a and Myf5.
(A) 3D chromatin conformation models of the Mef2a locus generated from pCHi-C data from ESC, +Dox and –Dox iPax7 cells. The top-scoring 3D models in each population are shown. (B) Distance distribution between Mef2a promoter (highlighted in blue) and any marker (active enhancer and SE as shown on top of the line plot) for each cell population. Line plot at 5kb resolution displays the median distance distribution between the Mef2a promoter and all the particles containing a CTCF peak or an active enhancer in the ensemble of models of ESC (blue, n=486), +Dox (green, n=940) and -Dox (orange, n=946). The bar displayed for most of the dots indicates one standard deviation from the distribution median. (C) Genome browser tracks around the Myf5 locus (the promoter is highlighted in yellow), showing ChIP-seq, ATAC-seq, RNA-seq, Pax7 binding sites, pCHi-C probes and high-confidence pCHi-C interactions (magenta arcs with associated virtual 4C plots) in ESC and iPax7 cells before (+Dox) and after (-Dox) differentiation. SE is only detected in Dox-treated iPax7 cells. (D) Distance distributions between enhancers and their closest TSS (Pax7 En: n=2,756; All En: n=9,380) versus pCHi-C-captured promoters (observed; Pax7 En: n=4,237; All En: n=15,012). Pax7 En, Pax7 enhancers with P-En interactions. All En, all enhancers with P-En interactions detected in +Dox iPax7 cells. Boxplots show the 25th and 75th percentile (bottom and top of box), and median value (horizontal band inside box). The whiskers indicate the values observed within up to 1.5 times the interquartile range above and below the box. Statistical significance tested with two-tailed Student's t-test. the interquartile range, and whiskers show 5th and 95th percentile. Asterisks indicate statistical significance (p<0.001) tested with two-tailed Student's t-test. (E) CTCF and cohesin co-localization at the two anchors of Pax7-associated P-En interactions.

SupFig.8

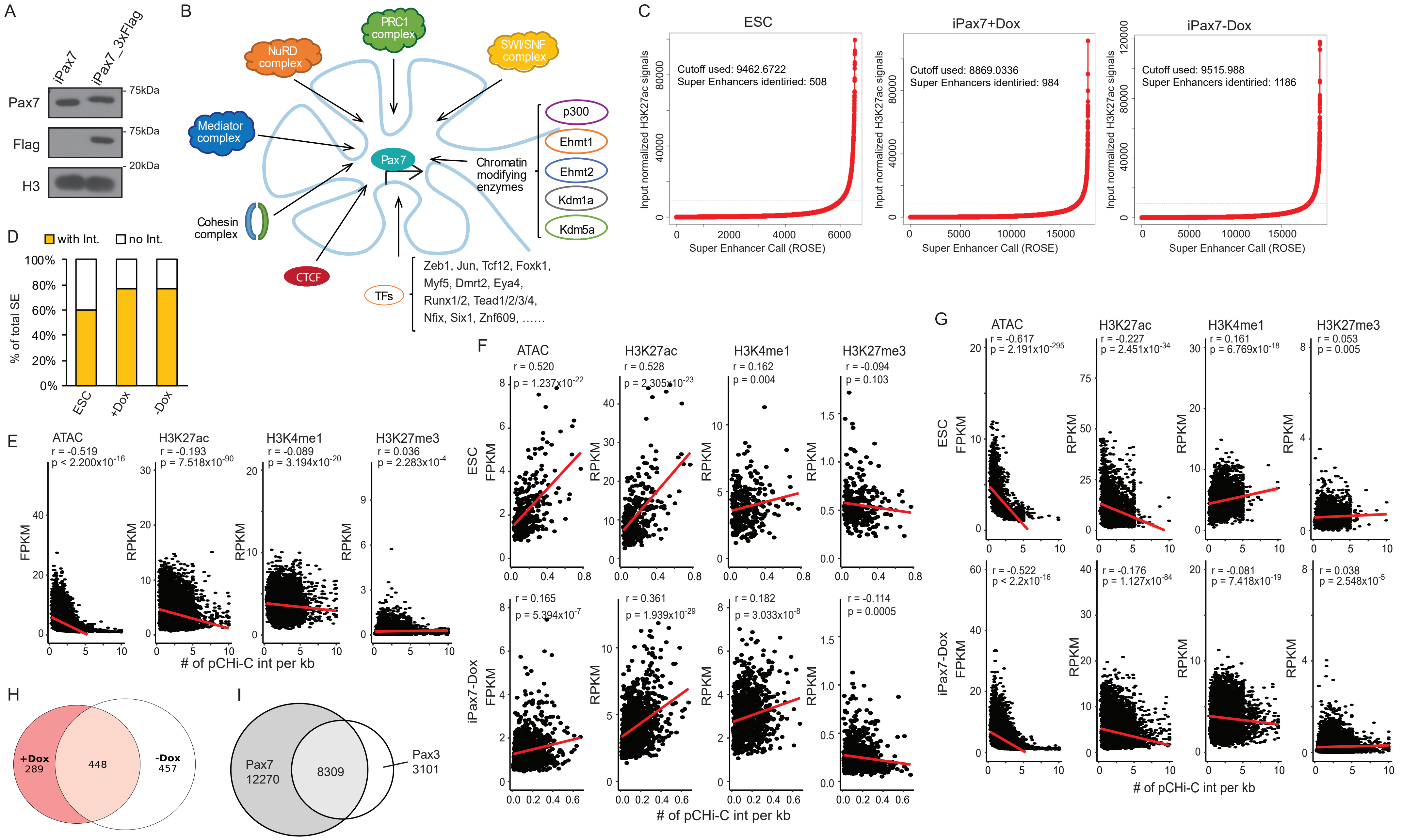
A
iPax7
iPax7_3xFlag
Pax7
Flag
H3
75kDa
75kDa
20kDa
B
PRC1 complex
NuRD complex
SWI/SNF complex
Mediator complex
Pax7
Chromatin modifying enzymes
p300
Ehmt1
Ehmt2
Kdm1a
Kdm5a
Cohesin complex
CTCF
TFs
Zeb1, Jun, Tcf12, Foxk1,
Myf5, Dmrt2, Eya4,
Runx1/2, Tead1/2/3/4,
Nfix, Six1, Znf609, ……
C
ESC
Cutoff used: 9462.6722
Super Enhancers identiried: 508
iPax7+Dox
Cutoff used: 8869.0336
Super Enhancers identiried: 984
iPax7-Dox
Cutoff used: 9515.988
Super Enhancers identiried: 1186
Input normalized H3K27ac signals
Super Enhancer Call (ROSE)
D
with Int.
no Int.
% of total SE
ESC
+Dox
-Dox
E
ATAC
r = -0.519
p < 2.200x10-16
H3K27ac
r = -0.193
p = 7.518x10-90
H3K4me1
r = -0.089
p = 3.194x10-20
H3K27me3
r = 0.036
p = 2.283x10-4
FPKM
RPKM
# of pCHi-C int per kb
F
ATAC
r = 0.520
p = 1.237x10-22
H3K27ac
r = 0.528
p = 2.305x10-23
H3K4me1
r = 0.162
p = 0.004
H3K27me3
r = -0.094
p = 0.103
ESC
r = 0.165
p = 5.394x10-7
r = 0.361
p = 1.939x10-29
r = 0.182
p = 3.033x10-8
r = -0.114
p = 0.0005
iPax7-Dox
# of pCHi-C int per kb
G
ATAC
r = -0.617
p = 2.191x10-295
H3K27ac
r = -0.227
p = 2.451x10-34
H3K4me1
r = 0.161
p = 6.769x10-18
H3K27me3
r = 0.053
p = 0.005
ESC
r = -0.522
p < 2.2x10-16
r = -0.176
p = 1.127x10-84
r = -0.081
p = 7.418x10-19
r = 0.038
p = 2.548x10-5
iPax7-Dox
# of pCHi-C int per kb
H
+Dox
289
448
-Dox
457
I
Pax7
12270
8309
Pax3
3101

Supplementary Figure 8. Identification of Pax7-interacting proteins and promoter-SE interactions in ESC, +Dox and –Dox iPax7 cells.

(A) Immuno-blot for nuclear extracts of Dox treated iPax7 and Flag-tagged iPax7 cells. (B) Summary of our proteomic screen showing selected Pax7-interacting proteins and complexes enriched in Dox-treated iPax7 cells versus control cells lacking Dox. (C) Super-enhancer calling using H3K27ac ChIP-seq data in ESC, +Dox and -Dox iPax7 cells. x-axis shows the rank of enhancers, and larger number represents higher rank of peaks with H3K27ac signals. y-axis is the input normalized H3K27ac ChIP-seq coverage within the peak. (D) Quantification of SEs with and without pCHiC interactions in ESC (n=508), +Dox (n=984) and -Dox (n=1,186) iPax7 cells. (E) Pairwise Pearson correlation between the number of significant pCHi-C interactions per kb and signals for ATAC-seq and ChIP-seq for histone modifications at individual enhancers in iPax7 muscle progenitors. A linear regression line (y ~ x) is plotted in red. r: Pearson correlation coefficient. p values determined by two-tailed Student's t-test. (F, G) Pairwise Pearson correlation between the number of significant pCHi-C interactions per kb and signals for ATAC-seq and ChIP-seq for histone modifications at SEs (F) and individual enhancers (G) in ESC and iPax7 cells without Dox (-Dox). A linear regression line (y ~ x) is plotted in red. r: Pearson correlation coefficient. p values determined by two-tailed Student's t-test. (H) Overlap between SEs with high-confidence pCHiC interactions in +Dox and -Dox iPax7 cells. (I) Overlap between Pax7 and Pax3 ChIP-seq peaks in ESC derived inducible myogenic precursors.

SupFig.9

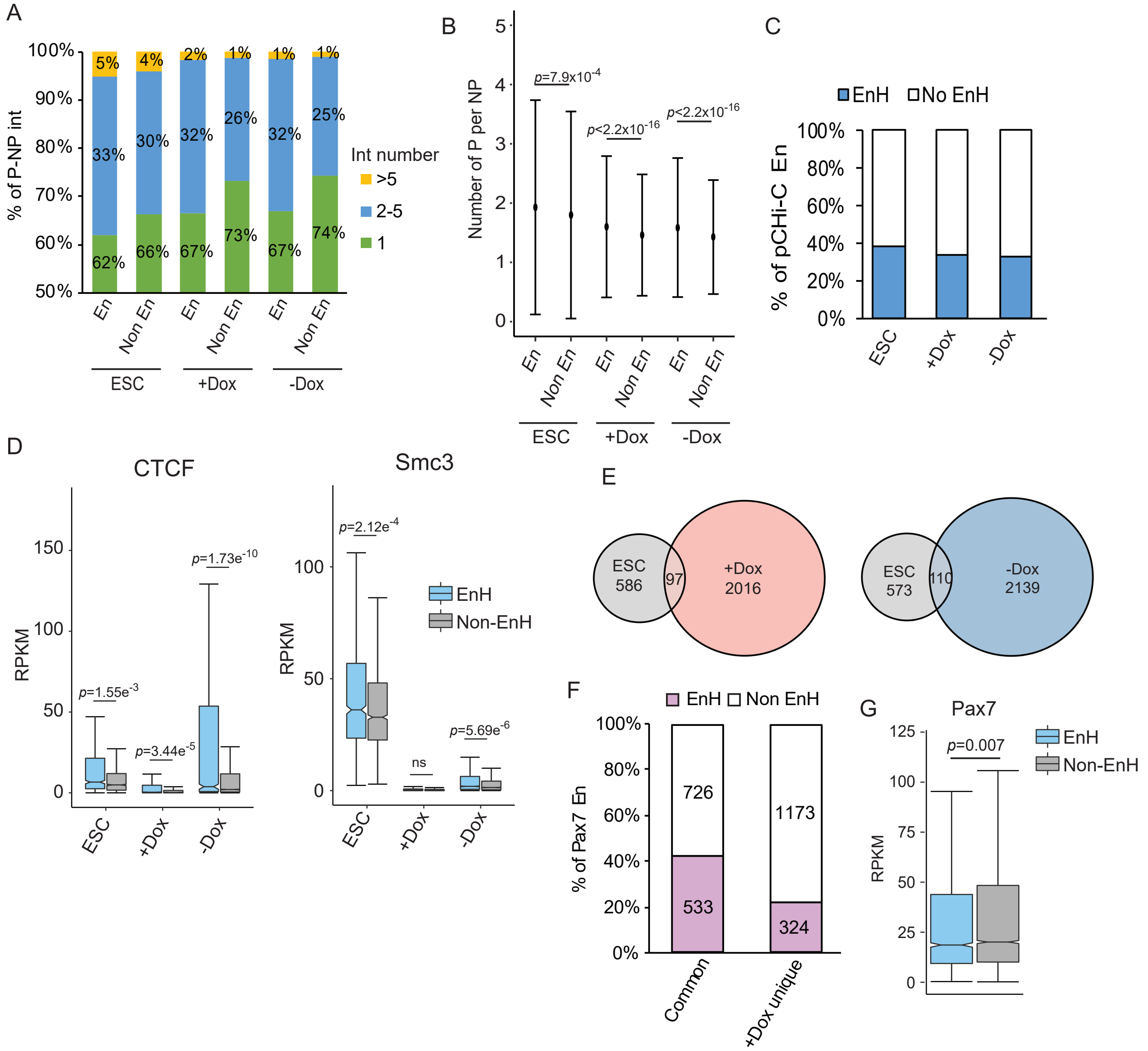

Supplementary Figure 9. Characterizing EnH in ESC and iPax7 cells.
(A) Quantification of promoter-non-promoter (P-NP) interactions for active enhancers (En) and non-enhancer (Non En) regions in ESCs (En: n=2,325; non En: n=31,190) and iPax7 cells (for +Dox, En: n=9,380; non En: n=41,763; for –Dox, En: 10,452; non En: 38,167). High-confidence P-NP interactions are categorized by the number of interactions connecting to each non-promoter region. (B) Average number (dot) of pCHiC promoters (P) per non-promoter regions (NP) interacting with enhancer and non-enhancer regions in ESCs (En: n=2,325; non En: n=31,190)ESC and iPax7 cells (for +Dox, En: n=9,380; non En: n=41,763; for –Dox, En: 10,452; non En: 38,167). Error bars indicate s.d., and statistical results determined by two-tailed Student's t-test. (C) Quantification of EnHs among all pCHiC captured enhancer regions in ESCs (n=1,989) and iPax7 cells (+Dox: n=7,701; -Dox: n=8,672). (D) CTCF and Smc3 ChIP-seq signal enrichment at EnH and non-ECH enhancers in ESC (EnH: n=683; Non-EnH: n=1,306), +Dox (EnH: n=2,113; Non-EnH: n=5,588) and -Dox (EnH: n=2,249; Non-EnH: n=6,423) iPax7 cells. The boxes denote the 25th and 75th percentile (bottom and top of box), and median value (horizontal band inside box). The whiskers indicate the values observed within up to 1.5 times the interquartile range above and below the box. Statistical significance: two-tailed Student's t-test. (E) Overlaps between EnHs in ESC and iPax7 cells with (top) and without (bottom) Dox. (F) Quantification of EnHs among Pax7 enhancers that are involved in common and +Dox unique P-En interactions. (G) Enrichment of Pax7 ChIP-seq signal at EnH and non-EnH enhancers identified in +Dox iPax7 cells (EnH: n=2,113; Non-EnH: n=5,588). The boxes denote the 25th and 75th percentile (bottom and top of box), and median value (horizontal band inside box). The whiskers indicate the values observed within up to 1.5 times the interquartile range above and below the box. Statistical significance tested with two-tailed Student's t-test.

Supplementary Table 1. List of all sequencing data used in this study.

Deposited Data	Citation	Data access
Data Generated in This Study		
Raw and analyzed data (Hi-C, pCHi-C, and anti-CTCF and anti-Smc3 ChIP-seq in iPax7 cells (+Dox & -Dox), and ATAC-seq in iPax7 ESCs (-Dox))	This paper	GSE150638
anti-Pax7_ChIP-seq_iPax7+Dox	This paper	GSE147057
Published Data Used in This Study		
ChIP-seq		
anti-Runx1_primary myoblast	Umansky et al., 2015	GSE56077
anti-Flag_Flag-Six4_C2C12	Chakroun et al., 2015	GSE66901
anti-Tead1_C2C12_undiff	Joshi et al., 2017	GSE82193
anti-Tead4_C2C12_undiff	Joshi et al., 2017	GSE82193
anti-Foxk1_C2C12	Bowman et al., 2014	GSE56932
anti-Jun_C2C12	Blum et al., 2012	GSE37525
anti-MyoD_C2C12	Mouse ENCODE functional genomics data	GSE36024
anti-MyoG_C2C12_60h	Mouse ENCODE functional genomics data	GSE36024
anti-Cebpb_C2C12	Mouse ENCODE functional genomics data	GSE36024
anti-Fosl1_C2C12	Mouse ENCODE functional genomics data	GSE36024
anti-Tcf3_C2C12_5d	Mouse ENCODE functional genomics data	GSE36024
anti-Tcf12_C2C12_60h	Mouse ENCODE functional genomics data	GSE36024
anti-E2F4_C2C12_60h	Mouse ENCODE functional genomics data	GSE36024
anti-CTCF_ESC	Shen et al., 2012	GSE29184
anti-Smc3_ESC	Shen et al., 2012	GSE29184
anti-H3K4me1_ESC	Shen et al., 2012	GSE29184
anti-H3K4me3_ESC	Shen et al., 2012	GSE29184
anti-H3K27ac_ESC	Shen et al., 2012	GSE29184
anti-H3K27me3_ESC	Shen et al., 2012	GSE29184
anti-Pax3_6d_Dox_iPax3_EB	Magli et al., 2019	GSE125203
ATAC-seq		
ATAC_iPax7+Dox	Lilja et al., 2017	GSE89977
ATAC_iPax7-Dox_3d	Lilja et al., 2017	GSE89977
ATAC_C2C12_3xFlag	Lilja et al., 2017	GSE89977
ATAC_C2C12_Pax7_3xFlag	Lilja et al., 2017	GSE89977
RNA-seq		
RNA-seq_ESC	Shen et al., 2012	GSE29184
RNA-seq_iPax+Dox	Lilja et al., 2017	GSE89977
RNA-seq_iPax7-Dox_3d	Lilja et al., 2017	GSE89977
Hi-C		
ESC_HindIII	Dixon et al., 2012	GSE35156
pCHi-C		
ESC	Schoenfelder et al., 2015	E-MTAB-2414
3T3_L1_d0	Siersbæk et al., 2017	GSE95533
3T3_L1_4h	Siersbæk et al., 2017	GSE95533
3T3_L1_d2	Siersbæk et al., 2017	GSE95533

SupplementaryTable 2. CRISPRi sgRNA sequences and oligonucleotides for real-time PCR.

Control sgRNA oligo for CRISPRi	
sgCAG	GTTCCGCGTTACATAACTTA

sgRNA oligos for CRISPRi targeting *Dmrt2* enhancers	
sgEn1	ATGAGGCGGCACCGAGAGCTTGG
sgEn2	CATGGTCACTTAGCGAAAGCAGG
sgEn3	CGACTAATGCAAATTGTCACTGG

sgRNA oligos for CRISPRi targeting the *Myh* EnH	
sgEn1	TCAGCATAGCTCGGGGTAAC
sgEn2	GAGCGGTGGTGCCACCTAGT
sgEn3	TCACGTGACTTGATTCATGC

Real time PCR oligos	
Gapdh _F	CGGAGTCAACGGATTTGGTCGTAT
Gapdh _R	AGCCTTCTCCATGGTGGTGAAGAC
B2m _F	GTCGCTTCAGTCGTCAGCAT
B2m _R	TTGAGGGGTTTTCTGGATAGCA
Dmrt2 _F	AAAGCAGTGTACCAGAGGCA
Dmrt2 _R	AGGCGGAGGTAGAGGAAGG
Myod1 _F	AGCACTACAGTGGCGACTCA
Myod1 _R	GGCCGCTGTAATCCATCA
Myh1 _F	TTGACTTTGGGATGGACCTG
Myh1 _R	GCCTTAGGGAACATGCACTC
Myh3 _F	AAGAGTAGCCAGGATGGGAAA
Myh3 _R	CATGGCATACACGTCCTCTG
Myh8 _F	CAACCTCAAAGAGCGTTATGC
Myh8 _R	CAGCCACTTGTAGGGGTTGA

Supplementary Table 3. Genomic regions used for 3D modeling.

Cell	Loci	Spearman correlation	p-value
ESC	Mef2a	0.68018912	3.29E-76
ESC	Dmrt2	0.756774827	0.00E+00
ESC	Myod1	0.779541165	0.00E+00
minusDox	Mef2a	0.79559612	6.79E-123
minusDox	Dmrt2	0.819703042	0.00E+00
minusDox	Myod1	0.779547115	0.00E+00
plusDox	Mef2a	0.799458522	3.70E-125
plusDox	Dmrt2	0.817737522	0.00E+00
plusDox	Myod1	0.796050522	0.00E+00
