# Supplementary material for: Muscle progenitor specification and myogenic differentiation are associated with changes in chromatin topology
Source: Nat Commun. 2020 Dec 4;11:6222. doi: 10.1038/s41467-020-19999-w (PMC7718254; doi:10.1038/s41467-020-19999-w)
Supplement: Supplementary file 7 — Description of Additional Supplementary Files [file 41467_2020_19999_MOESM7_ESM.pdf]

Description of Additional Supplementary Files

Supplementary Data 1.
Alignment statistics of pCHi-C and Hi-C experiments.

Supplementary Data 2.
P-En interaction coordinates and target genes for each cluster in Fig. 2D.

Supplementary Data 3.
DESeq2 statistics for individual gene expression of four groups of Pax7 En target genes.

Supplementary Data 4.
Mass spectrometry detected Pax7 interacting proteins.
