# Supplementary material for: Muscle progenitor specification and myogenic differentiation are associated with changes in chromatin topology
Source: Nat Commun. 2020 Dec 4;11:6222. doi: 10.1038/s41467-020-19999-w (PMC7718254; doi:10.1038/s41467-020-19999-w)
Supplement: Supplementary file 8 — Reporting Summary [file 41467_2020_19999_MOESM8_ESM.pdf]

nature research

Corresponding author(s): Brian David Dynlacht

Last updated by author(s): Oct 31, 2020

Reporting Summary

Nature Research wishes to improve the reproducibility of the work that we publish. This form provides structure for consistency and transparency in reporting. For further information on Nature Research policies, see our Editorial Policies and the Editorial Policy Checklist.

Statistics

For all statistical analyses, confirm that the following items are present in the figure legend, table legend, main text, or Methods section.

n/a	Confirmed	
☐	☒	The exact sample size (*n*) for each experimental group/condition, given as a discrete number and unit of measurement
☐	☒	A statement on whether measurements were taken from distinct samples or whether the same sample was measured repeatedly
☐	☒	The statistical test(s) used AND whether they are one- or two-sided<br>*Only common tests should be described solely by name; describe more complex techniques in the Methods section.*
☐	☒	A description of all covariates tested
☐	☒	A description of any assumptions or corrections, such as tests of normality and adjustment for multiple comparisons
☐	☒	A full description of the statistical parameters including central tendency (e.g. means) or other basic estimates (e.g. regression coefficient) AND variation (e.g. standard deviation) or associated estimates of uncertainty (e.g. confidence intervals)
☐	☒	For null hypothesis testing, the test statistic (e.g. *F*, *t*, *r*) with confidence intervals, effect sizes, degrees of freedom and *P* value noted<br>*Give P values as exact values whenever suitable.*
☒	☐	For Bayesian analysis, information on the choice of priors and Markov chain Monte Carlo settings
☒	☐	For hierarchical and complex designs, identification of the appropriate level for tests and full reporting of outcomes
☐	☒	Estimates of effect sizes (e.g. Cohen's *d*, Pearson's *r*), indicating how they were calculated

Our web collection on statistics for biologists contains articles on many of the points above.

Software and code

Policy information about availability of computer code

Data collection	No software was used for data collection.
Data analysis	SAINT (Significance Analysis of Interactome)<br>HiC-bench<br>bowtie2<br>HOMER<br>HiCUP<br>Juicebox<br>CHiCAGO<br>WashU EpiGenome browser<br>Cytoscape version 3.7.2<br>Picard-tools version 1.88<br>DESeq2<br>STAR version 2.5.0c<br>DAVID version 6.8<br>MACS2 version 2.1.1<br>deepTools version 3.1.0<br>ROSE<br>MEME-ChIP<br>CRISPOR<br>TADbit

TADdyn
R version 3.6.3
samtools
bedtools
Chimera version 1.14
Proteome Discoverer version 1.4

For manuscripts utilizing custom algorithms or software that are central to the research but not yet described in published literature, software must be made available to editors and reviewers. We strongly encourage code deposition in a community repository (e.g. GitHub). See the Nature Research guidelines for submitting code & software for further information.

Data

Policy information about availability of data

All manuscripts must include a data availability statement. This statement should provide the following information, where applicable:

- Accession codes, unique identifiers, or web links for publicly available datasets
- A list of figures that have associated raw data
- A description of any restrictions on data availability

All NGS data generated in this study have been deposited in NCBI/GEO with the accession codes GSE150638 and GSE147057. Source data from previously published work (listed in Supplementary Table 1) are available under the following accession numbers: GSE56077, GSE66901, GSE82193, GSE56932, GSE37525, GSE36024, GSE29184, GSE125203, GSE89977, GSE35156 and GSE95533 from GEO and E-MTAB-2414 from the ArrayExpress database (http://www.ebi.ac.uk/arrayexpress). The mass spectrometry data have been deposited to the MassIVE Repository (https://massive.ucsd.edu/) with the dataset identifier MSV000086392.

Field-specific reporting

Please select the one below that is the best fit for your research. If you are not sure, read the appropriate sections before making your selection.

☒ Life sciences ☐ Behavioural & social sciences ☐ Ecological, evolutionary & environmental sciences

For a reference copy of the document with all sections, see nature.com/documents/nr-reporting-summary-flat.pdf

Life sciences study design

All studies must disclose on these points even when the disclosure is negative.

Sample size	No statistical method were used to determine sample size. For CRISPRi, western blot, immune-purification and mass spectrometric sequencing experiments, n>=3 was chosen to meet the minimal replicate number, and the cell number used for each experiment was determined by the standard protocols. We determined this to be sufficient owing to internal controls (expression of known reference genes/ proteins) and high reproducibility of the results.
Data exclusions	No data were excluded.
Replication	All NGS data generated in this study were from biological duplicates. Proteomic data were generated from biological triplicates. All other validation experiments were performed at least three times. We followed the same protocol to generate biological replicates for each of our experiments. And the analysis of the data were reliably reproduced.
Randomization	For NGS and proteomic experiments, all reads/peptides that passed quality control were analyzed equally with no sub-sampling and thus, there was no requirement for randomization.
Blinding	Not applicable since no specific grouping.

Reporting for specific materials, systems and methods

We require information from authors about some types of materials, experimental systems and methods used in many studies. Here, indicate whether each material, system or method listed is relevant to your study. If you are not sure if a list item applies to your research, read the appropriate section before selecting a response.

Materials & experimental systems

n/a	Involved in the study	
☐	☒	Antibodies
☐	☒	Eukaryotic cell lines
☒	☐	Palaeontology and archaeology
☒	☐	Animals and other organisms
☒	☐	Human research participants
☒	☐	Clinical data
☒	☐	Dual use research of concern

Methods

n/a	Involved in the study	
☐	☒	ChIP-seq
☒	☐	Flow cytometry
☒	☐	MRI-based neuroimaging

Antibodies

Antibodies used	anti-CTCF (Cat. 07-729, Millipore; dilution: 1:2,000), anti-Smc3 (Cat. ab9263, abcam; dilution: 1:2,000), anti-Flag (Cat. F7425, Sigma; dilution: 1:1,000), anti-H3 (Cat. ab1791, abcam; dilution: 1:1,000), anti-Pax7 (Developmental Studies Hybridoma Bank (DSHB); dilution: 1:500), and anti-Six1 (Cat. 10709-1-AP, Proteintech; dilution: 1:500).
Validation	All antibodies are from commercially available and have been used extensively. Below are links to these products.<br>anti-CTCF: https://www.emdmillipore.com/US/en/product/Anti-CTCF-Antibody,MM_NF-07-729<br>anti-Smc3: https://www.abcam.com/smc3-antibody-chip-grade-ab9263.html<br>anti-Flag: https://www.sigmaaldrich.com/catalog/product/sigma/f7425?lang=en&region=US<br>anti-H3: https://www.abcam.com/histone-h3-antibody-nuclear-loading-control-and-chip-grade-ab1791.html<br>anti-Pax7: https://dshb.biology.uiowa.edu/PAX7<br>anti-Six1: https://www.ptglab.com/products/SIX1-Antibody-10709-1-AP.htm

Eukaryotic cell lines

Policy information about cell lines

Cell line source(s)	iPax7 and iPax7_3xFlag cells (mouse, from R. C. R. Perlingeiro lab, see ref. 19, 20)
Authentication	Cell lines have been authenticated in previous publications cited in our manuscript.
Mycoplasma contamination	No evidence of mycoplasma contamination.
Commonly misidentified lines (See ICLAC register)	No commonly misidentified cell lines were used.

ChIP-seq

Data deposition

- [x] Confirm that both raw and final processed data have been deposited in a public database such as GEO.
- [x] Confirm that you have deposited or provided access to graph files (e.g. BED files) for the called peaks.

Data access links<br>*May remain private before publication.*	GSE147057: https://www.ncbi.nlm.nih.gov/geo/query/acc.cgi?acc=GSE147057<br>GSE150638: https://www.ncbi.nlm.nih.gov/geo/query/acc.cgi?acc=GSE150638
Files in database submission	Raw fastq or bam files:<br>ChIP_CTCF_iPax7+Dox_rep1<br>ChIP_CTCF_iPax7+Dox_rep2<br>ChIP_CTCF_iPax7-Dox_rep1<br>ChIP_CTCF_iPax7-Dox_rep2<br>ChIP_Smc3_iPax7+Dox_rep1<br>ChIP_Smc3_iPax7+Dox_rep2<br>ChIP_Smc3_iPax7-Dox_rep1<br>ChIP_Smc3_iPax7-Dox_rep2<br>ChIP_input_iPax7+Dox_rep1<br>ChIP_input_iPax7+Dox_rep2<br>ChIP_input_iPax7-Dox_rep1<br>ChIP_input_iPax7-Dox_rep2<br>Input_6day_dox_rep1 (Pax7 control)<br>Input_6day_dox_rep2 (Pax7 control)<br>ChIPseq_6day_dox_EB_rep1 (Pax7)<br>ChIPseq_6day_dox_EB_rep2 (Pax7)<br><br>Processed files:<br>GSE150638_iPax7+Dox_CTCF.merge.bigWig<br>GSE150638_iPax7+Dox_CTCF_peaks.narrowPeak.gz<br>GSE150638_iPax7+Dox_Smc3.merge.bigWig<br>GSE150638_iPax7+Dox_Smc3_peaks.narrowPeak.gz<br>GSE150638_iPax7+Dox_input.merge.bigWig<br>GSE150638_iPax7-Dox_CTCF.merge.bigWig<br>GSE150638_iPax7-Dox_CTCF_peaks.narrowPeak.gz<br>GSE150638_iPax7-Dox_Smc3.merge.bigWig<br>GSE150638_iPax7-Dox_Smc3_peaks.narrowPeak.gz<br>GSE150638_iPax7-Dox_input.merge.bigWig<br>GSE147057_mm9_Pax7_q01_peaks.narrowPeak.gz

Genome browser session (e.g. UCSC)	None

Methodology

Replicates	All newly generated ChIP-seq experiments were repeated twice.
Sequencing depth	ChIP_CTCF_iPax7+Dox_rep1, paired-end, total 23448105 reads, uniquely mapped 9361802 reads, read length is 37 bp.<br>ChIP_CTCF_iPax7+Dox_rep2, paired-end, total 19204095 reads, uniquely mapped 4354556 reads, read length is 37 bp.<br>ChIP_CTCF_iPax7-Dox_rep1, paired-end, total 25394417 reads, uniquely mapped 23571234 reads, read length is 37 bp.<br>ChIP_CTCF_iPax7-Dox_rep2, paired-end, total 24019820 reads, uniquely mapped 18760028 reads, read length is 37 bp.<br>ChIP_Smc3_iPax7+Dox_rep1, paired-end, total 20092000 reads, uniquely mapped 3444164 reads, read length is 37 bp.<br>ChIP_Smc3_iPax7+Dox_rep2, paired-end, total 23208526 reads, uniquely mapped 6451788 reads, read length is 37 bp.<br>ChIP_Smc3_iPax7-Dox_rep1, paired-end, total 15,216,522 reads, uniquely mapped 7123174 reads, read length is 37 bp.<br>ChIP_Smc3_iPax7-Dox_rep2, paired-end, total 26687521 reads, uniquely mapped 15113976 reads, read length is 37 bp.<br>ChIP_input_iPax7+Dox_rep1, paired-end, total 29861143 reads, uniquely mapped 5579240 reads, read length is 37 bp.<br>ChIP_input_iPax7+Dox_rep2, paired-end, total 34652325 reads, uniquely mapped 6674032 reads, read length is 37 bp.<br>ChIP_input_iPax7-Dox_rep1, paired-end, total 24729310 reads, uniquely mapped 13943432 reads, read length is 37 bp.<br>ChIP_input_iPax7-Dox_rep2, paired-end, total 34458494 reads, uniquely mapped 25197646 reads, read length is 37 bp.<br>Input_6day_dox_rep1 (Pax7 control), single-end, total reads 23469320, uniquely mapped 13445760 reads, read length is 50 bp.<br>Input_6day_dox_rep2 (Pax7 control), single-end, total reads 46382201, uniquely mapped 26589319 reads, read length is 50 bp.<br>ChIPseq_6day_dox_EB_rep1 (Pax7), single-end, total 37977339 reads, uniquely mapped 22796343 reads, read length is 50 bp.<br>ChIPseq_6day_dox_EB_rep2 (Pax7), single-end, total 36115252 reads, uniquely mapped 20895761 reads, read length is 50 bp.
Antibodies	anti-CTCF (Cat. 07-729, Millipore), anti-Smc3 (Cat. ab9263, abcam) and Pax7 (Developmental Studies Hybridoma Bank).
Peak calling parameters	Peak-calling was done using MACS2 version 2.1.1 (macs2 callpeak -t $Treat -c $Contrl -f BAM -g mm -n ${Out}) with a default q-value of 0.05 for CTCF and Smc3 ChIP-seq and a q-value cut-off of 0.01 for Pax7 ChIP-seq.Replicate experiments were merged and the data were normalized per million total reads for visualization.
Data quality	A cut-off q-value of 0.05 for CTCF and Smc3 ChIP-seq and a q-value of 0.01 for Pax7 ChIP-seq were used for peak calling. Number of peaks at FDR 5% and above 5-fold enrichment for each sample is listed as follows:<br>ChIP_CTCF_iPax7+Dox (merged): 70598 peaks;<br>ChIP_CTCF_iPax7-Dox (merged): 92180 peaks;<br>ChIP_Smc3_iPax7+Dox (merged): 59213 peaks;<br>ChIP_Smc3_iPax7-Dox (merged): 76246 peaks;<br>Pax7 ChIP-seq (merged): 20579 peaks.
Software	Raw reads were aligned to the mouse genome version NCBIM37/mm9 with bowtie2 version 2.3.4.1 (specific settings for paired-end reads: --local --no-mixed --no-discordant; specific settings for single-end reads: --local). PCR duplicates were removed using Picard-tools version 1.88. Peak-calling was done using MACS2 version 2.1.1. Heatmaps and profile plots for normalized ChIP-seq data were generated using deepTools version 3.1.0.
